# *BRC1* expression regulates bud activation potential but is not necessary or sufficient for bud growth inhibition in *Arabidopsis*

**DOI:** 10.1242/dev.145649

**Published:** 2017-05-01

**Authors:** Madeleine Seale, Tom Bennett, Ottoline Leyser

**Affiliations:** Sainsbury Laboratory, University of Cambridge, Bateman Street, Cambridge CB2 1LR, UK

**Keywords:** Shoot branching, *BRANCHED1*, Bud growth inhibition, Auxin, Strigolactone

## Abstract

The degree of shoot branching in *Arabidopsis* is determined by the activation of axillary buds. Bud activity is regulated by diverse environmental and developmental signals, often mediated via plant hormones, including auxin, strigolactone and cytokinin. The transcription factor *BRANCHED1* (*BRC1*) has been proposed to integrate these regulatory signals. This idea is based on increased branching in *brc1* mutants, the effects of bud-regulating hormones on *BRC1* expression, and a general correlation between *BRC1* expression and bud growth inhibition. These data demonstrate the important role of *BRC1* in shoot branching, but here we show that in *Arabidopsis* this correlation can be broken. Buds lacking *BRC1* expression can remain inhibited and sensitive to inhibition by strigolactone. Furthermore, buds with high *BRC1* transcript levels can be active. Based on these data, we propose that *BRC1* regulates bud activation potential in concert with an auxin transport-based mechanism underpinning bud activity. In the context of strigolactone-mediated bud regulation, our data suggest a coherent feed-forward loop in which strigolactone treatment reduces the probability of bud activation by parallel effects on *BRC1* transcription and the shoot auxin transport network.

## Abstract (precis)

**Summary:** BRC1 is neither necessary nor sufficient for bud growth inhibition in a variety of contexts; instead, it likely modulates bud activation potential within a wider system of bud activity control.

## INTRODUCTION

The degree of shoot branching is an excellent example of plant developmental plasticity. In flowering plants, secondary shoots are formed by the activation of axillary buds, established in the axils of leaves produced by active shoot apical meristems. Differential activation of axillary buds allows a continuum of shoot forms, from a solitary unbranched stem to a highly ramified bush. Bud activity is regulated by diverse endogenous and environmental inputs. The integration of these inputs shapes the shoot system according to genotype and environment.

The mechanisms by which buds integrate regulatory signals are poorly understood. There are two non-exclusive candidates for the integrating hub (Rameau et al., 2015). According to the auxin transport canalization-based model for bud activation, sustained bud activity requires the establishment of canalized auxin transport from the bud into the main stem (Balla et al., 2011; Bennett et al., 2006; Li and Bangerth, 1999, 2003; Prusinkiewicz et al., 2009). The auxin transport canalization hypothesis posits that an initial passive flux of auxin between an auxin source and an auxin sink results in the upregulation and polarization of auxin transporters in the direction of the flux, canalizing auxin transport into files of cells with highly polar, high-capacity auxin transport connecting the auxin source to the sink (Sachs, 1969; 1981; see also Bennett et al., 2014). Axillary buds are potential auxin sources, and the main stem acts as an auxin sink by transporting auxin rootwards (Prusinkiewicz et al., 2009; Sachs, 1968). As all active apices export auxin into the stem, thereby reducing its sink strength, axillary buds effectively compete for access to a common auxin transport path down the stem to the root. The ability of a bud to activate is therefore relative, depending on its strength as an auxin source compared with the auxin sink strength of the stem, and on the degree of positive feedback between auxin flux and auxin transporter upregulation/polarization. This system can act as a hub for the integration of multiple signals, which could influence bud activity by modulating stem auxin sink strength, bud auxin source strength or canalization feedback.

The canalization-based model of bud regulation can account for diverse shoot branching phenomena, including apical dominance. It is long established that auxin produced by the growing primary apex is transported down the stem, inhibiting the activity of subtending axillary buds (Thimann and Skoog, 1934). Decapitation removes the auxin source, allowing sustained bud growth, which is prevented by application of auxin to the decapitated stump (Thimann and Skoog, 1934). This auxin acts indirectly because very little is transported into the bud itself (Booker et al., 2003; Brown et al., 1979; Everat-Bourbouloux and Bonnemain, 1980; Prasad et al., 1993). Restated in terms of the canalization hypothesis, apically derived auxin in the stem inhibits sustained bud activation by reducing stem auxin sink strength, hence preventing auxin export from buds. The canalization-based model can also explain how the plant hormone strigolactone (SL) inhibits branching. One effect of SL is to trigger removal of the PIN-FORMED1 (PIN1) auxin exporter from the plasma membrane, thereby dampening positive feedback between auxin flux and auxin transporter polarization/upregulation, making it more difficult for buds to activate (Crawford et al., 2010; Prusinkiewicz et al., 2009; Shinohara et al., 2013). This can explain why SL treatment enhances competition between branches on a two-node stem segment, focusing growth into one branch, rather than simply inhibiting both branches (Crawford et al., 2010).

The other likely regulatory hub for bud activity is expression of TCP transcription factors of the *TEOSINTE BRANCHED1* (*TB1*) class. *TB1* was originally identified through its role in maize domestication (Doebley et al., 1997). In contrast to its wild relative teosinte, maize is unbranched. This is due to constitutive overexpression of *TB1* in maize, which inhibits the activity of axillary buds, but also influences floral transition in branches (Hubbard et al., 2002). As a result, branches from the middle nodes of the maize primary stem develop and terminate as female inflorescences (ears), whereas in teosinte they develop as elongating branches.

Closely related genes have been characterised in several species, including *FINE CULM1* (*FC1*) in rice and *BRANCHED1* (*BRC1*) in *Arabidopsis*, pea, tomato and potato (Aguilar-Martínez et al., 2007; Arite et al., 2007; Braun et al., 2012; Dun et al., 2012; Finlayson, 2007; Takeda et al., 2003; Martín-Trillo et al., 2011; Nicolas et al., 2015). In core eudicots, there appear to be three co-homologues of *TB1/FC1* from grasses (Citerne et al., 2013; Howarth and Donoghue, 2006; Martín-Trillo and Cubas, 2010). In *Arabidopsis* two of these genes, *BRC1* and *BRC2*, have effects on branching, but it is *BRC1* that has the major effect and is proposed to act as a regulatory hub (Aguilar-Martínez et al., 2007; Poza-Carrión et al., 2007). Similar to *TB1*, *BRC1* also affects aspects of the floral transition (Aguilar-Martínez et al., 2007; Niwa et al., 2013).

Across these species, loss of function of *TB1* class genes results in increased branching. Furthermore, their transcript levels correlate with bud growth inhibition and change rapidly in response to bud-regulatory treatments. It has therefore been proposed that the transcription of *TB1* class genes acts as an integrated read-out of bud-regulatory signals. For example, in pea, the level of *BRC1* transcript in buds is regulated positively by the bud-activating hormone cytokinin (CK), and negatively by SL, with both bud activity and *BRC1* transcript abundance reaching intermediate levels when buds are treated with both hormones simultaneously (Braun et al., 2012; Dun et al., 2012). In this model, apical dominance is attributed to the ability of auxin to increase expression of SL biosynthetic genes and reduce the expression of CK biosynthetic genes in the stem, thereby regulating the supply of these hormones to buds (Dun et al., 2012, 2013). Consistent with this idea, the buds of *brc1* mutants of both pea and *Arabidopsis* have been reported to be completely insensitive to SL (Brewer et al., 2009; Braun et al., 2012).

These two models are not mutually exclusive, but they are distinct. SL signalling/synthesis mutants have both increased PIN1 accumulation in the shoot and decreased bud *BRC1* transcript levels, but these effects are apparently independent (Bennett et al., 2016b). Beyond this, it has proved difficult to design experiments that distinguish between the two models. It has not been possible to separate sustained bud outgrowth from bud auxin export, although limited bud elongation can occur in the presence of auxin transport inhibitors (Brewer et al., 2015). However, interpretation of these results is problematic because, as described above, the absolute levels of auxin transport are of limited relevance to bud activation, and it is difficult to make clean local perturbations that affect the relevant relative properties of the system. Thus, the observation that plants with systemically inhibited auxin transport can still respond to strigolactone is consistent with both modes of action for strigolactone (Brewer et al., 2015). This problem is exacerbated by the fact that a central tenet of auxin transport canalization is that auxin transporters polarize in a flux-correlated manner and, despite its demonstrable predictive power, this process is currently entirely mechanistically obscure, making it difficult to perturb the system in informative ways (Bennett et al., 2014).

Conversely, there are unambiguous examples in which the correlation between bud growth inhibition and *BRC1* transcript levels is broken. For example, maize SL-deficient mutants are highly branched but maintain constitutively high *TB1* expression (Guan et al., 2012), and genetic variation in maize branching does not correlate with *TB1* expression (Kebrom and Brutnell, 2015). Furthermore, in rice, *FC1* transcript abundance is apparently insensitive to SL (Arite et al., 2007; Minakuchi et al., 2010). In addition, in *Psbrc1* and *fc1* mutants, some buds remain inhibited (Arite et al., 2007; Braun et al., 2012). These data suggest that *BRC1* cannot be a simple branch regulatory hub. In order to explore this issue further, we have focused on the role of *BRC1* in *Arabidopsis* bud growth inhibition, where many relevant tools are available. We show that *BRC1* is neither necessary nor sufficient for bud growth inhibition in a variety of contexts. Our data suggest that *BRC1* acts to modulate bud activation potential, within a wider system of bud activity control.

## RESULTS

### *BRC1* transcript abundance decreases as buds activate

Following floral transition, *Arabidopsis* buds activate in a basipetal sequence starting at the uppermost cauline (inflorescence) node, proceeding down the shoot into the rosette nodes (Alvarez et al., 1992; Hempel and Feldman, 1994). We have previously shown that buds remain inactive when a young inflorescence, bearing one or two leaves, is removed from the plant and placed in a microcentrifuge tube containing nutrient medium (Ongaro et al., 2008; Prusinkiewicz et al., 2009). The buds can be activated by decapitation of the primary shoot apex (Ongaro et al., 2008) or basal supply of CK (Müller et al., 2015). To characterise the dynamics of *BRC1* transcript abundance in this system, we quantified *BRC1* transcript levels and the initiation of bud elongation in response to bud-activating treatments.

**Fig. 1.**
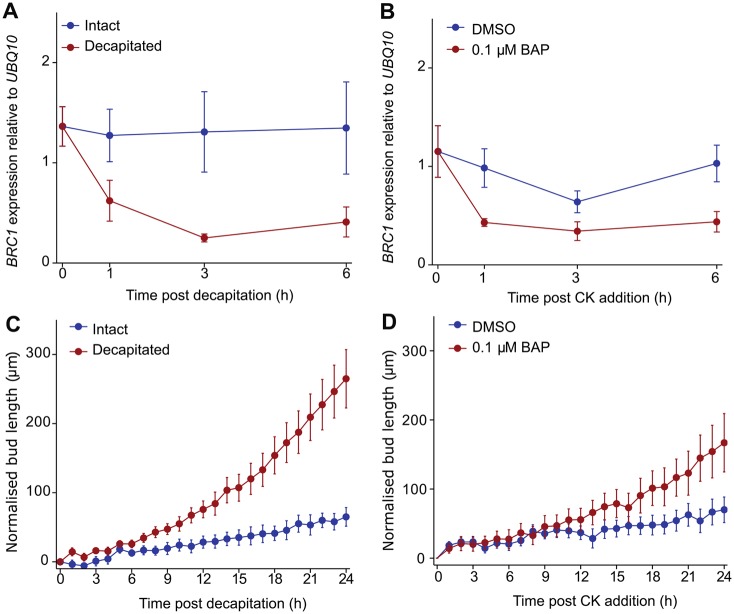
**Levels of** ***BRC1* transcripts decrease rapidly after bud activation.** (A,B) Transcript levels of *BRC1* in pooled samples of buds from 15-20 individual one-node explants. Each panel shows the mean of three to five independent biological repeats, error bars are s.e.m. (A) 6 h time course of buds with intact primary shoot apex or activated by decapitation; (B) 6 h time course of buds with DMSO control treatment or activated by 0.1 µM basal benzylaminopurine (BAP), a synthetic CK. (C,D) Mean length of the bud stem over time, *n*=9-12, error bars are s.e.m. (C) Buds activated by decapitation or with an intact primary shoot apex; (D) buds activated with 0.1 µM basal BAP treatment or DMSO controls.

Excised shoot apices bearing a single cauline node with its associated axillary bud were left untreated, decapitated directly above the node or supplied basally with CK [0.1 µM benzylaminopurine (BAP)]. A decrease in bud *BRC1* transcript abundance was detected 1 h after decapitation, and levels continued to drop until 3 h post-decapitation (Fig. 1A). Decreases in *BRC1* transcripts were not detected at 15 or 30 min post-decapitation (Fig. S1). Similarly, *BRC1* transcripts decreased in buds within 1 h of basal CK treatment (Fig. 1B). To correlate this with bud growth, time-lapse images of treated and control buds were captured every hour for 24 h, and the length of the elongating bud stem, between the axil and the oldest bud leaf, was determined. Control buds elongate slowly across this time course (Fig. 1C). However, decapitation activates rapid bud growth well within 24 h. After log transformation of the data to allow parametric testing, significant differences in bud stem length between intact and decapitated explants were apparent from 8 h (*P*<0.05 for independent two-sample *t*-tests between treatment groups from 8 h). With basal CK treatment, rapid growth begins later (Fig. 1D), with a significant CK effect not observed until 19 h after treatment (*P*<0.05 for independent two-sample *t*-tests between treatment groups from 19 h).

Accurate bud stem measurement is challenging as growing buds slightly change their orientation within the focal plane of the camera. Given this, and more generally the likely differences in sensitivity of qRT-PCR versus length measurements, it cannot be clearly established whether changes in *BRC1* transcript abundance and growth are simultaneous, or one precedes the other. Interestingly, although both bud-activating treatments trigger a significant drop in *BRC1* transcript abundance within 1 h, significant differences in bud length are detected 11 h later for CK-activated buds compared with decapitation-activated buds.

### Bud *BRC1* transcript abundance responds to an apical signal

**Fig. 2.**
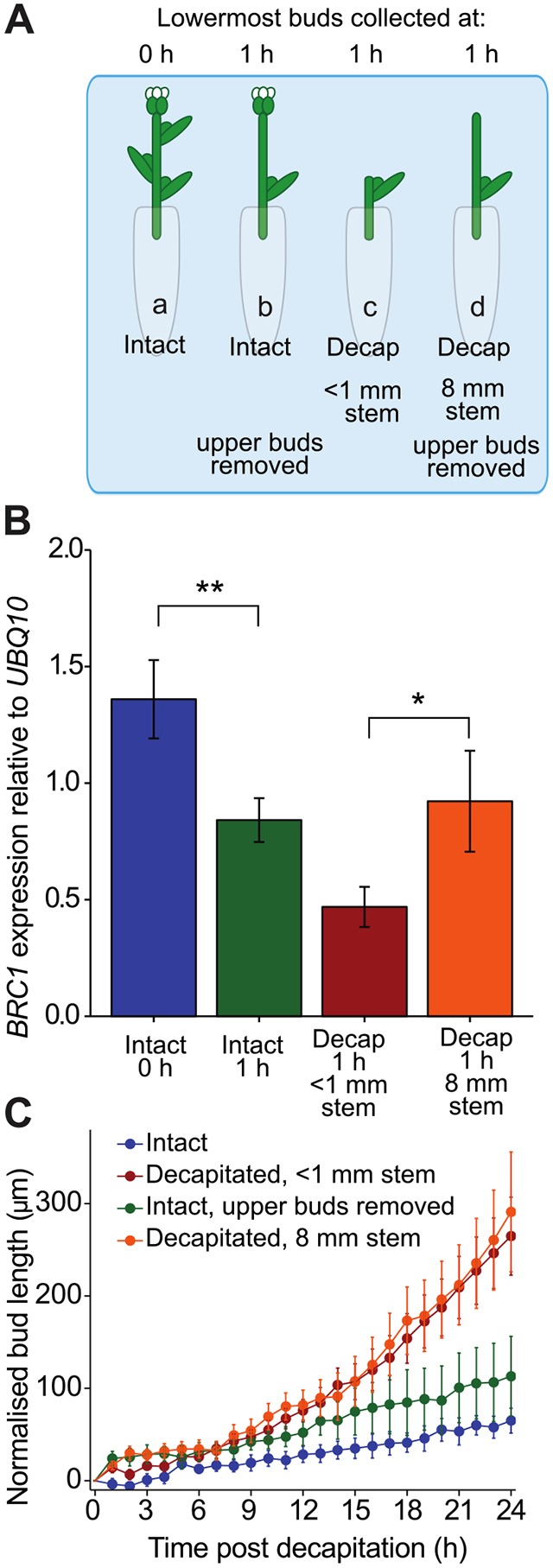
***BRC1* transcript levels respond to an apical signal.** (A) Experimental setup: manipulation of the length of main stem above the node following decapitation. In treatment a, the lowermost buds were collected before decapitation; in b, the lowermost buds were collected 1 h after removal of intermediate buds; in c, buds were collected 1 h after decapitation directly above the lowermost bud; in d, lowermost buds were collected 1 h after decapitation and removal of upper buds, leaving 8 mm of stem. (B) *BRC1* transcript levels 0 h and 1 h after decapitation with treatments according to A; results are the mean of four independent biological repeats with 15-20 buds per sample, error bars are s.e.m. Asterisks indicate a statistically significant difference between groups with two-sample *t*-tests and Holm-Bonferroni adjustment at *P<0.1 or **P<0.05. (C) Length of the bud stem of buds with intact or decapitated primary shoot apex with treatments according to A. ‘Intact’ and ‘Decapitated <1 mm stem’ are the same data reproduced from Fig. 1C, error bars are s.e.m. *n*=9-12.

An obvious hypothesis is that decapitation triggers changes in *BRC1* transcript levels through main stem auxin depletion. To manipulate the time at which a bud might experience changes in main stem auxin, the length of stem between the bud and the decapitation site was varied (Bennett et al., 2016a). Stem explants bearing three nodes were isolated and subjected to four different treatments: intact primary shoot apex with lowest buds collected at 0 h; intact primary shoot apex with intermediate buds removed and lowest buds collected at 1 h; lowest buds collected 1 h after decapitation directly above the bud; and lowest buds collected 1 h after decapitation 8 mm above the bud, with intermediate buds removed (Fig. 2A).

As expected, 1 h after decapitation immediately above the bud, a substantial reduction in *BRC1* transcript abundance was observed. Decapitation 8 mm above the bud also reduced *BRC1* transcript levels, but they were significantly higher than those observed in buds decapitated immediately above the node (Fig. 2B). Interestingly, in intact explants from which the intermediate buds had been removed, there was also a small but significant reduction in *BRC1* transcripts in the lower bud relative to the 0 h time point. This suggests that even inactive buds can influence *BRC1* transcription in the other buds in the shoot system, e.g. by contributing a small amount of auxin to the main stem. Together, these data suggest that an apically derived signal, such as auxin, moving basipetally in the stem regulates *BRC1* transcription.

To determine whether decapitation 8 mm above the bud also delays bud activation, bud stem elongation was assessed using time-lapse imaging. There was no detectable difference in the timing of bud stem elongation between explants decapitated directly versus 8 mm above the bud (Fig. 2C). Additionally, removing the upper buds of intact explants did not significantly alter bud length. Thus, the subtle differences in *BRC1* transcript dynamics observed in this assay are not reflected in detectable differences in bud elongation, although this could be because of the differential sensitivity of these assays, as described above.

### Bud *BRC1* transcript abundance responds to auxin in the main stem

**Fig. 3.**
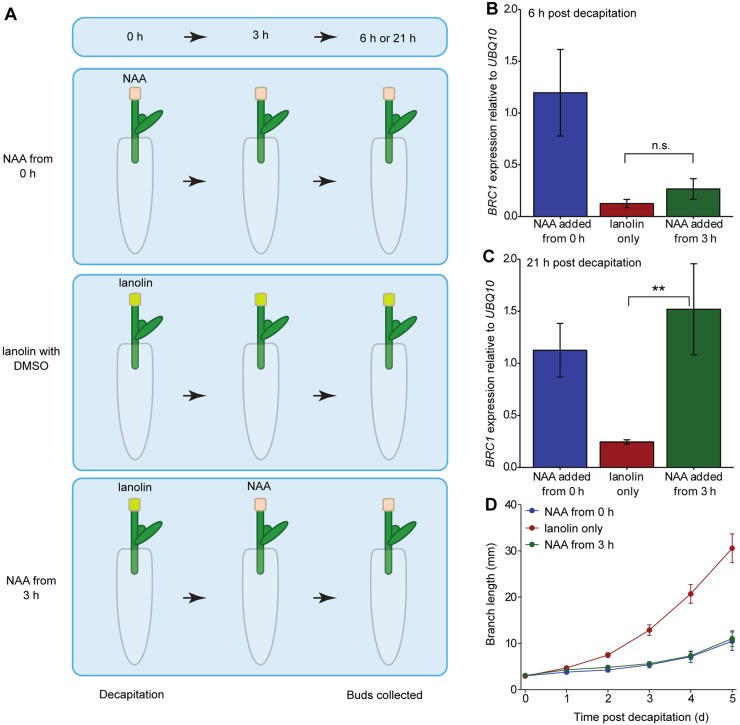
***BRC1* transcript levels respond to apical auxin.** (A) Experimental setup: explants bearing single nodes were decapitated and 1 mM NAA (1-naphthylene acetic acid, a synthetic auxin) in lanolin or the control treatment was placed on decapitated stump at either 0 h or 3 h post-decapitation. Buds were collected 6 or 21 h post-decapitation. (B,C) *BRC1* transcript levels in pooled bud samples of 15-20 explants treated according to A collected at 6 h post-decapitation (B) or 21 h post-decapitation (C). Brackets indicate the results of two-sample *t*-tests as not significant (n.s.) or significant at **P<0.05. (D) Bud length over time for each treatment, *n*=14-16. The data are representative of three independent biological repeats, error bars are s.e.m.

To assess more directly whether main stem auxin influences bud *BRC1* transcript abundance, a similar experimental setup was used, but with auxin applied to the decapitated stump in lanolin paste. Explants were decapitated immediately above the node and subjected to one of three apical auxin treatments (Fig. 3A): lanolin containing the auxin analogue 1-naphthylene acetic acid (NAA) (1 mM); lanolin with dimethyl sulphoxide (DMSO); or lanolin with DMSO for the first 3 h after decapitation then replaced with lanolin containing 1 mM NAA. Buds were collected 6 h (Fig. 3B) or 21 h (Fig. 3C) after the start of the experiment, giving the ‘NAA from 3 h’ explants 3 h or 18 h of apical NAA, respectively.

When apical auxin was supplied immediately after decapitation, *BRC1* transcript abundance remained high (Fig. 3B,C). By contrast, the ‘NAA from 3 h’ buds showed a major reduction in *BRC1* transcript levels at 6 h post-decapitation, to levels not significantly different from the lanolin-only buds (Fig. 3B). By 21 h post-decapitation, *BRC1* transcript abundance in ‘NAA from 3 h’ buds returned to levels comparable with those in ‘NAA from 0 h’ buds, and significantly higher than the lanolin-only control (Fig. 3C). Both apical auxin treatments prevented bud outgrowth compared with lanolin-only controls (Fig. 3D), despite the transient dip in *BRC1* transcript abundance in ‘NAA from 3 h’ buds. Together, these data indicate that bud *BRC1* transcripts are dynamically responsive to auxin in the main stem, but only sustained downregulation of *BRC1* is associated with sustained bud outgrowth.

### Auxin-mediated bud inhibition is partially dependent on *BRC1*

**Fig. 4.**
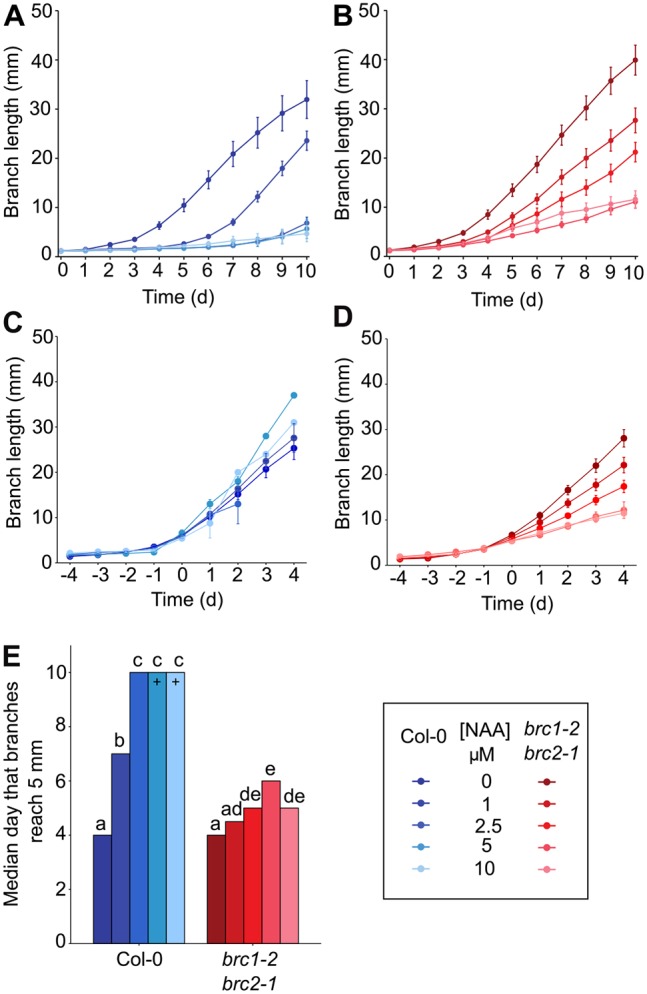
***brc1-2 brc2-1* buds have altered auxin responses.** Branch lengths of isolated one-node explants of (A) wild-type Col-0 and (B) *brc1-2 brc2-1* held between two agar slabs with apical NAA at concentrations of 1, 2.5, 5 and 10 µM, or ethanol only. Bud length was measured for 10 days after excision of explants, *n*=19-20, error bars are s.e.m. Data are representative of two independent repeats. (C) Wild-type and (D) *brc1-2 brc2-1* growth curves of the same data aligned to the activation day (day 0) defined as the day on which branches reached 5 mm or greater. Some explants do not feature at some treatments and/or time-points as they never reached 5 mm, or did so late in the experiment, giving sample sizes between 1 and 20. (E) Median day on which buds reach 5 mm or more in length. Crosses indicate a median of at least 10 days, with insufficient buds reaching lengths greater than 5 mm for these treatments. Different letters above bars indicate statistically signiﬁcant differences at *P*<0.05, with log-rank test with Holm-Bonferroni adjustment. Comparisons were carried out between genotypes of the same treatment and between treatments of the same genotype.

To investigate the relationship between auxin-mediated bud inhibition and *BRC1* activity, we tested the sensitivity of *brc1-2 brc2-1* mutant buds to apical auxin supply. Plants were grown axenically and stem segments bearing one cauline node with an associated bud less than 1.5 mm were excised and inserted between two agar slabs in a Petri dish (Chatfield et al., 2000). Auxin was applied via the apical slab at concentrations ranging from 1 to 10 µM and bud growth was monitored for 10 days (Fig. 4A,B).

In wild type, apical auxin delayed bud activation, as shown by plotting the median number of days for buds to reach 5 mm, which increased with increasing auxin concentration (Fig. 4E). However, even with high auxin treatments, a few buds eventually activated, elongating at a rate similar to untreated buds. This can be seen by aligning the growth curves for each bud at the time-point when the bud first reached or exceeded 5 mm (defined as t=0 on the graph in Fig. 4C). Thus, in wild type, auxin delays bud activation without affecting its kinetics.

The buds of *brc1-2 brc2-1* mutants responded differently to apical auxin (Fig. 4B,D). They did not show the typical switching behaviour seen in wild type, but either elongated slowly throughout the experiment, or elongated slowly and then arrested (Fig. 4B). The onset of elongation was only slightly delayed, even by high concentrations of auxin (Fig. 4E), but the subsequent rate of elongation was inversely proportional to auxin concentration (Fig. 4D). These data suggest that *BRC1* is involved in modulating bud activation dynamics in response to auxin.

### *BRC1* is not necessary for bud growth inhibition

**Fig. 5.**
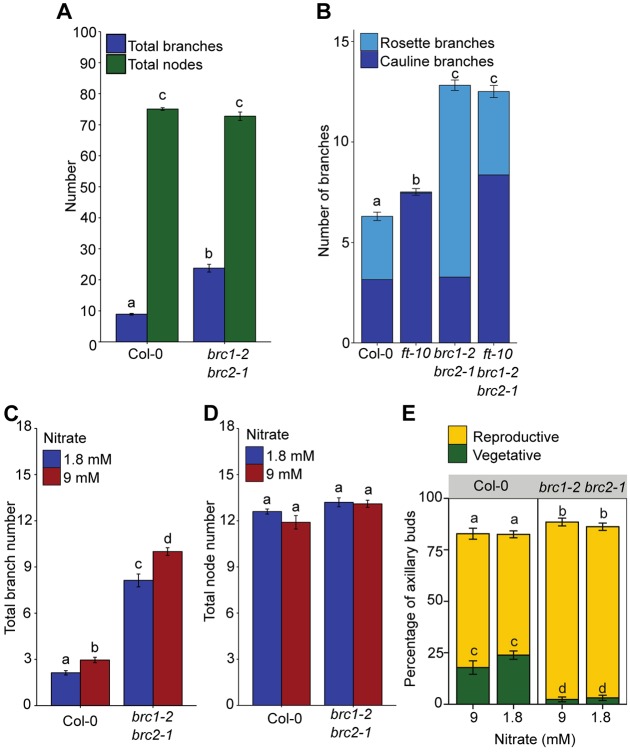
***BRC1* is not necessary for bud growth inhibition.** Branch numbers and vegetative node numbers scored at proliferative arrest. (A) Total number of branches and nodes for plants grown in short days, *n*=16-23, representative of two independent repeats. (B) Number of rosette and cauline branches in wild-type, *ft-10*, *brc1-2 brc2-1* and *ft-10 brc1-2 brc2-1* plants, *n*=39-40, representative of two independent repeats. (C) Number of branches, *n*=21-23, representative of three independent repeats, and (D) number of nodes, *n*=10, on plants grown with high (9 mM) or low (1.8 mM) nitrate supply. (E) Percentage of nodes with axillary buds that were vegetative or reproductive in plants grown with high or low nitrate supply, *n*=10. Different letters above bars indicate statistically significant differences at *P*<0.05, with Wilcoxon rank-sum tests with Holm-Bonferroni adjustments where appropriate. Error bars are s.e.m.

In long-day-grown *Arabidopsis*, the number of primary branches formed in *brc1-2 brc2-1* closely matches the number of primary axis leaves (Aguilar-Martínez et al., 2007; Bennett et al., 2016b). This could indicate that *BRC1* is essential for bud growth inhibition. To test this idea, we used low nitrate availability, which suppresses branching in *Arabidopsis* (de Jong et al., 2014). Plants were grown with high (9 mM) or low (1.8 mM) nitrate supply. As expected, wild-type plants produced significantly more branches on high compared with low nitrate (Fig. 5C). Although *brc1-2 brc2*-*1* mutants had significantly more branches than wild type in both conditions, they were still capable of reducing branch number under nitrate limitation (Fig. 5C). As previously reported (Aguilar-Martínez et al., 2007; Niwa et al., 2013), *brc1-2 brc2-1* plants had a greater proportion of floral branches in both conditions compared with wild type (Fig. 5E). However, there were no differences in the total number of primary leaf-bearing nodes either between genotypes or between treatments, and *brc1-2 brc2-1* mutants had inhibited buds in both conditions (Fig. 5C,D). These data demonstrate that *BRC1* is not necessary for bud growth inhibition.

To investigate this effect further in a situation with a larger number of primary leaf-bearing nodes, and therefore buds, we grew wild-type and *brc1-2 brc2-1* mutants in short-day growth conditions and determined the number of primary leaf-bearing nodes and branches formed. The number of primary leaves was similar between the two genotypes (Fig. 5A). While *brc1-2 brc2-1* still produced twice as many branches as wild type, more than half its nodes did not produce an elongated branch (Fig. 5A), confirming that *BRC1* is not necessary for bud growth inhibition.

To manipulate leaf number in long-day conditions, we crossed *brc1-2 brc2-1* into the *flowering locus t-10* (*ft-10*) mutant background, which has delayed flowering and increased vegetative node number when grown in long-day growth conditions (Koornneef et al., 1991; Yoo et al., 2005). We observed that the total number of branches on wild-type and *ft-10* plants was similar (Fig. 5B). Likewise, the number of branches produced by *brc1-2 brc2-1* and *ft-10 brc1-2 brc2-1* plants was almost identical (Fig. 5B). Thus, as in short days, *brc1-2 brc2-1* double mutants have many inhibited buds in the *ft-10* background. Loss of *BRC1* increases the number of buds that activate, but this effect is limited and independent of the total number of leaves, and hence buds, on the primary axis. It has previously been shown that *brc1* has accelerated floral transition in its branches, and that this is suppressed by loss of *FT* (Niwa et al., 2013). Our data suggest that *ft* does not suppress the *brc1* branch outgrowth phenotype (Fig. 5B).

### *BRC1* transcript accumulation is not sufficient for bud growth inhibition

**Fig. 6.**
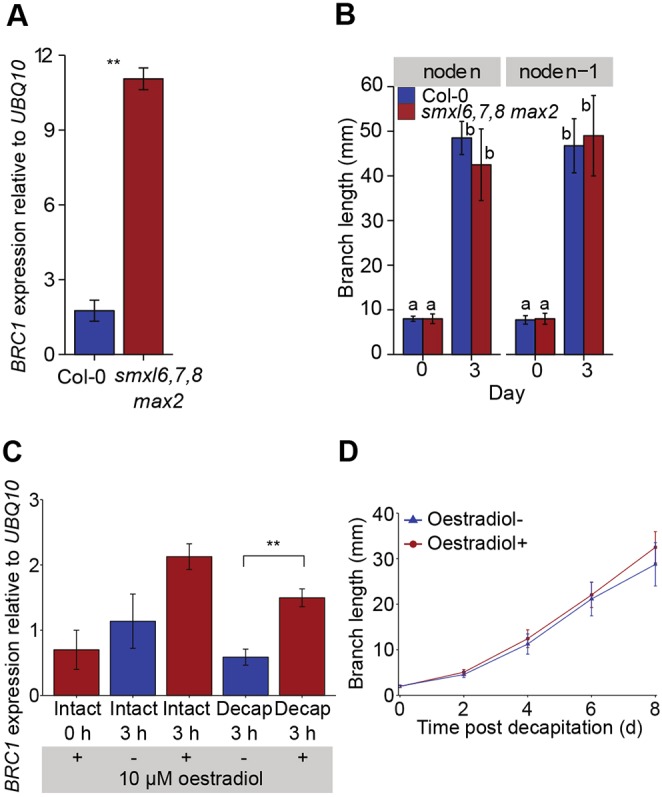
**High levels of bud *BRC1* transcripts do not prevent branch growth.** (A) *BRC1* transcript levels in the two apical-most buds (n and n-1) of wild type and *smxl678 max2* mutants. Pooled bud samples were taken from four plants per genotype. **P<0.05. (B) Branch length of apical n and n-1 branches of wild type and *smxl678 max2* mutants 0 and 3 days after decapitation, *n*=4. Different letters above bars indicate statistically significant differences between groups with pairwise *t*-tests and Holm-Bonferroni adjustment. (C,D) Oestradiol-inducible *lexa::BRC1* explants treated basally with 10 µM β-oestradiol for 90 min before decapitation. (C) *BRC1* transcript levels in buds of induced and mock-treated explants. **P<0.05. (D) Branch length of induced and mock-treated explants after decapitation over time, *n*=10-15. For A and C, data are the mean of three biological replicates. Brackets with two asterisks indicate a statistically significant difference at *P*<0.05 with a two-sample *t*-test. For B and D, data are representative of two and three independent repeats, respectively. Error bars are s.e.m.

We next investigated whether high *BRC1* transcription is sufficient to prevent bud outgrowth. An oestradiol-inducible *lexa::BRC1* line was used to induce overexpression of *BRC1* (González-Grandío et al., 2013) by supplying 10 µM β-oestradiol basally to shoot explants bearing a single cauline node held in microcentrifuge tubes and either decapitated or left intact. After a 3 h treatment, oestradiol increased *BRC1* transcripts in both intact and decapitated samples to levels greater than normally observed in intact explants, where buds are inhibited (Fig. 6C). Specifically, *BRC1* transcript levels were significantly higher in induced versus uninduced decapitated samples, but this had no effect on branch growth (Fig. 6D). Branches grew normally, despite sustained contact with the oestradiol. Although it is possible that the high levels of *BRC1* were not sustained, the result suggests that *BRC1* transcript accumulation may not be sufficient to maintain bud growth inhibition.

To investigate situations in intact plants where growing buds might have high *BRC1* transcript levels, we turned to mutants in the recently identified SMAX1-LIKE6 (SMXL6), SMXL7 and SMXL8 proteins, which are the proteolytic targets of MAX2-mediated SL signalling (Soundappan et al., 2015; Wang et al., 2015). Their over-accumulation in SL signalling mutants, such as *max2*, results in increased branching and constitutively low *BRC1* transcript accumulation in buds. Mutation of *SMXL6*, *SMXL7* and *SMXL8* completely restores *max2* branching to wild type, and results in very high *BRC1* transcript levels in inhibited buds (Soundappan et al., 2015; Wang et al., 2015). This supports the hypothesis that SL inhibits bud growth by upregulating *BRC1* transcription. Despite this, the *smxl6-4 smxl7-3 smxl8-1 max2-1* (hereafter *smxl678 max2*) mutant has near wild-type levels of branching. We reasoned that *BRC1* expression might be constitutively high in *smxl678 max2* buds, whether active or inhibited. To test this hypothesis, buds 5-10 mm long (i.e. beginning to grow) from four plants of each genotype were pooled and their RNA extracted for analysis. In these apical *smxl678 max2* buds, *BRC1* transcript levels were extremely high compared with wild type (Fig. 6A). Despite this, these apical branches on *smxl678 max2* mutants grow with normal kinetics (Fig. 6B). This provides further evidence that high *BRC1* expression is not sufficient to suppress bud growth, although it is possible that there are post-transcriptional effects on BRC1 activity operating specifically in these buds.

### *brc1* is epistatic to *smxl6*, *smxl7* and *smxl8*

**Fig. 7.**
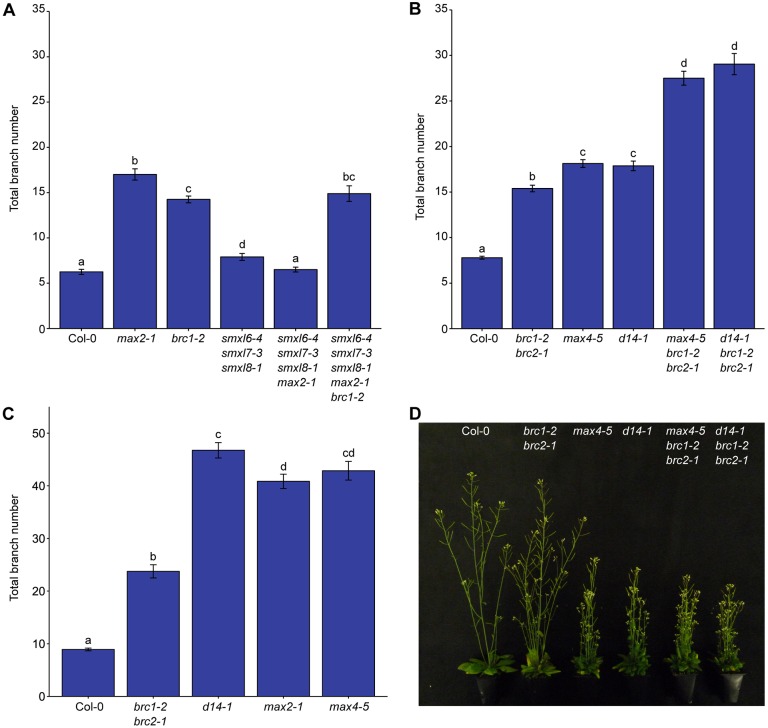
**Strigolactone and *BRC1* affect branching additively.** Total branch number at proliferative arrest. (A) Wild-type, *max2-1*, *brc1-2*, *smxl6-4 smxl7-3 smxl8-1*, *smxl6-4 smxl7-3 smxl8-1 max2-1* and *smxl6-4 smxl7-3 smxl8-1 max2-1 brc1-2* plants grown in long-day growth chambers, *n*=9-13. (B) Wild-type, *brc1-2 brc2-1*, *max4-5*, *d14-1*, *max4-5 brc1-2 brc2-1* and *d14-1 brc1-2 brc2-1* plants grown under long-day glasshouse conditions, *n*=23-24, representative of three independent repeats. (C) Wild-type, *brc1-2 brc2-1*, *d14-1*, *max2-1* and *max4-5* plants grown in short-day growth conditions, *n*=16-23, data for Col-0 and *brc1-2 brc2-1* are the same as for Fig. 5A. (D) Photographs of representative plants of the genotypes indicated grown for long days. Different letters above bars indicate statistically significant differences at *P*<0.05, with pairwise Wilcoxon rank-sum tests with Holm-Bonferroni adjustments, error bars are s.e.m.

This raises interesting questions about the relationship between strigolactone, *BRC1* and the *SMXL678* clade in the regulation of shoot branching. We therefore generated *smxl678 max2-1 brc1-2* quintuple mutants, and compared their branching with that of *smxl678 max2-1* quadruple mutants. Branching relationships among the parental types were as previously reported (Soundappan et al., 2015; Wang et al., 2015; Aguilar-Martínez et al., 2007). The *smxl678 max2-1* quadruple mutant had branching levels similar to wild type, with the highly branched phenotype of *max2* being completely suppressed (Fig. 7A). Strikingly *brc1-2* is epistatic to *smxl678* (Fig. 7A), bringing the branching level of *smxl678 max2-1* up to that of *brc1*. This result is consistent with a model in which SMXL678 promote branching by downregulating *BRC1*, but it is equally consistent with *BRC1* acting independently of SMXL678.

### *BRC1* is not required for strigolactone-mediated bud inhibition

Previous comparisons of *brc1* and SL signalling mutants showed that loss of SL signalling results in higher branching levels than loss of *BRC1* (Chevalier et al., 2014; Bennett et al., 2016b), suggesting that misregulation of *BRC1* expression is insufficient to explain the full branching phenotype of SL mutants. Extending this result, we found that growth in short days exaggerates the differences between SL mutants and *brc1-2 brc2-1*, with *max4-5*, *d14-1* and *max2-1* having almost double the branch number of *brc1-2 brc2-1* (Fig. 7C). We also made triple mutants between *max4-5* or *d14-1* and *brc1-2 brc2-1*. Consistent with previous results, the triple mutants had considerably more branches than either parent (Fig. 7B,D) (Chevalier et al., 2014). As SL mutants typically make a branch at almost every primary node, the strong additivity suggests activation of higher order branches in the triple mutant. The triple mutants also showed additivity with respect to the parental reduced height phenotypes (Fig. S2). These data provide further evidence that SL and *BRC1* act at least partially independently.

**Fig. 8.**
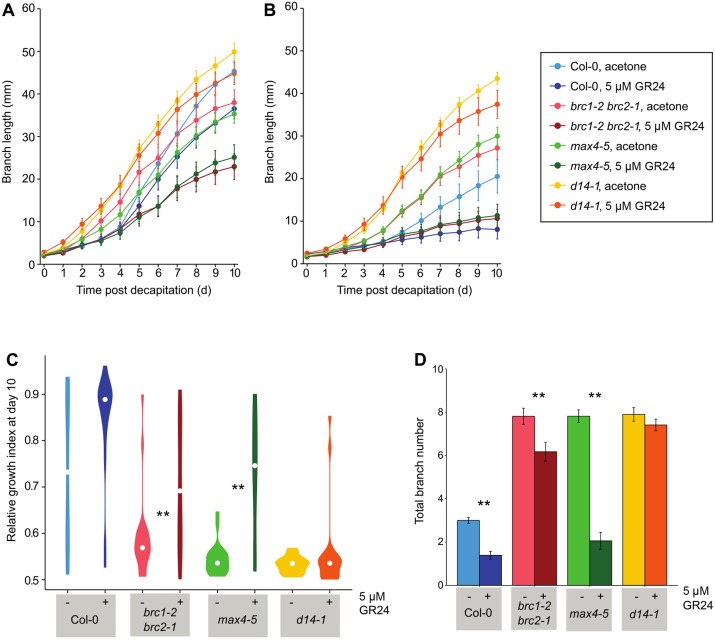
***brc1 brc2* mutants are strigolactone sensitive.** (A-C) Bud-bud competition assay in which explants bearing two nodes are basally treated with 5 µM GR24 (a synthetic strigolactone) or acetone control and decapitated, *n*=19-20, error bars are s.e.m. Length of the longer branch (A) or the shorter branch (B) over time after decapitation. (C) Violin plots of the relative growth index (the length of the longest branch divided by the total length of both branches) at day 10 after decapitation, white circles indicate the median and coloured shapes indicate the probability distribution of the data. (D) Total branch number of 6-week-old plants grown axenically on media supplemented with 5 µM GR24 or acetone control. For C and D, asterisks indicate statistically significant differences between treatments for each genotype at *P*<0.05 with pairwise Wilcoxon rank-sum tests. Data for all panels are representative of three independent repeats.

Support for the idea that SL regulates branching via *BRC1* comes from reports that *brc1* buds are SL insensitive (Brewer et al., 2009). Branching in *brc1* mutants was unaffected by growth on 5.8 µM GR24 (a strigolactone analogue). However, in the growth conditions used in that experiment very few branches were formed for any genotype regardless of treatment (Brewer et al., 2009). To explore SL response in more branch-conducive conditions, we grew plants of relevant genotypes axenically in jars on agar-solidified nutrient media supplemented with 5 µM GR24 or with a solvent control (Fig. 8D). The total number of branches longer than 1 cm was counted after 6 weeks (Fig. 8D). Consistent with previous reports (Crawford et al., 2010; Shinohara et al., 2013), GR24 strongly reduced branching in the SL biosynthesis mutant, *max4-5*, but had no effect on the SL signalling mutant, *d14-1*. Interestingly, branching in *brc1-2 brc2-1* was significantly reduced by GR24 treatment, though less than in *max4-5*, implying that *brc1-2 brc2-1* retains partial SL sensitivity.

To test this further, we used a bud-bud competition assay. Shoot explants bearing two cauline nodes were placed in microcentrifuge tubes with media supplemented with 5 µM GR24 or a control. The explants were decapitated and branch length measured over time, mimicking the classical two-branch pea assay developed by Snow (Snow, 1925, 1929, 1931; Ongaro et al., 2008). Typically in these experiments, both buds begin to grow, and over time either both continue to grow at a similar rate, or one dominates the other, leading to highly asymmetric growth of the two branches (Ongaro et al., 2008). The relative frequency of these outcomes is assessed using a relative growth index (RGI), defined as the length of the longest branch divided by the total length of both branches. A value close to 0.5 indicates equal branch growth, whereas values closer to 1 indicate that one branch dominates the other. In this assay, SL mutants have low RGIs compared with wild type, and GR24 treatment increases the RGI in wild-type and SL biosynthetic mutants, but not in SL signalling mutants (Fig. 8A-C), (Crawford et al., 2010). Untreated *brc1-2 brc2-1* explants had a low RGI, similar to *d14-1* and *max4-5* (Fig. 8C). GR24 treatment resulted in a significant increase in RGI, of a magnitude similar to that observed in wild-type and *max4-5* explants. In general, the phenotype of *brc1-2 brc2-1* in this assay was both quantitatively and qualitatively similar to *max4-5*. When comparing the mean length of the longer (Fig. 8A) and shorter (Fig. 8B) branches on each explant, it is clear that GR24 treatment strongly inhibits one branch in wild type, *max4-5*, and *brc1-2 brc2-1* mutants, while the other branch grows relatively vigorously. By contrast, both branches of the *d14-1* mutant grow strongly regardless of GR24 treatment. Therefore, in this assay, *brc1-2 brc2-1* mutants are fully strigolactone sensitive.

## DISCUSSION

### *BRC1* is not necessary or sufficient for bud growth inhibition

The *Arabidopsis BRC1* gene, and its homologues in other species, are important regulators of shoot branching. Their loss of function results in highly branched phenotypes and their expression correlates with bud growth inhibition. Indeed, *BRC1* transcript level is often used as a marker for bud growth inhibition. This has led to the hypothesis that branch regulatory signals are integrated at the level of *BRC1* expression (Aguilar-Martínez et al., 2007; Braun et al., 2012). The data we present here suggest that this integration operates within a wider framework for bud activity control. Consistent with previous results, we show that a reduction in *BRC1* transcripts is an early event during bud activation by decapitation or by CK treatment (Fig. 1), and that bud outgrowth is associated with a sustained reduction in *BRC1* transcripts (Aguilar-Martínez et al., 2007; Martín-Trillo et al., 2011; Braun et al., 2012; Nicolas et al., 2015). However, we also show that *brc1 brc2* mutants have inhibited buds (Fig. 5), and that elevated *BRC1* expression is not sufficient to prevent bud outgrowth (Fig. 6). Thus, high *BRC1* transcript levels are neither necessary nor sufficient for bud inhibition.

### A bud activation threshold model for *BRC1* action

As described above, after the floral transition in *Arabidopsis*, buds activate in a basipetal sequence. This sequence is likely generated by release of buds from auxin-mediated apical dominance, which is weakened at floral transition due to reduced auxin export from floral compared with vegetative apices (Prusinkiewicz et al., 2009). It is striking that the buds that remain inhibited in *brc1-2 brc2-1* mutants are those at the basal end of this sequence, whereas those that are active even with high *BRC1* transcription, as in the *smx678 max2* mutant, are those at the apical end. This suggests the hypothesis that *BRC1* modulates a bud activation threshold within the broader context of an auxin-mediated bud regulatory programme, such as the well-supported canalization-based mechanism described in the Introduction. According to this idea, for buds with high *BRC1* expression, activation requires a highly canalization-conducive environment, such as the relatively low stem auxin concentration at apical nodes. By contrast, for *brc1-2 brc2-1* buds, which lack *BRC1* expression, bud inhibition requires a strongly canalization-inhibitory environment, such as the high main stem auxin concentration at the basal nodes (Prusinkiewicz et al., 2009). This model could explain the correlation between bud inhibition and *BRC1* expression, while at the same time being fully consistent with the evidence that *BRC1* is neither necessary nor sufficient for bud inhibition. The model also straightforwardly explains why *brc1-2 brc2-1* mutants produce similar numbers of branches, irrespective of the number of leaf-bearing nodes on the primary stem (Fig. 5).

### *BRC1*, bud activation potential and shoot system architecture

Our data support the hypothesis that *BRC1* acts to modulate a bud activation threshold in concert with a systemic and relative auxin transport-mediated regulatory system for branching. *BRC1* transcript levels can be locally regulated by specific environmental cues, such as light quality (Finlayson et al., 2010; González-Grandío et al., 2013, 2017). Under our model, this would adjust the local activation threshold, allowing branches to activate (or be inhibited) out of the normal basipetal sequence. Thus, *BRC1* may be a point of integration between local, absolute regulatory inputs and systemic relative branching regulatory mechanisms, combining the advantages of both. In this way, *BRC1*-mediated variation in bud activation potential could also contribute to the patterns of branching observed in some species. For example, in rice the *BRC1* homologue *FC1* is expressed at higher levels in the most basal bud compared with the next bud up and this correlates with their growth inhibition (Arite et al., 2007).

This threshold model is also interesting in the context of recent evidence that changes in sugar accumulation in buds correlate strongly with the downregulation of *BRC1* expression and initiation of bud expansion in pea following decapitation (Mason et al., 2014). This effect can explain the initiation of bud expansion significantly ahead of main stem auxin depletion following decapitation. However, while application of apical auxin to the decapitated stump does not prevent this initial expansion, it does prevent sustained bud growth (Morris et al., 2005), despite the presumably high sugar content in the buds and the lack of a strong apical sugar sink. These observations are consistent with the idea that downregulation of *BRC1* can prime buds for activation, but this is not sufficient for sustained bud growth, which appears to require a canalization-permissive environment.

### Feed-forward regulation and bud activation dynamics

Although changes in *BRC1* transcript levels are not required for changes in bud activity, *BRC1* expression is clearly dynamically regulated during bud activation in *Arabidopsis*. For example, *BRC1* expression is rapidly downregulated following decapitation, likely in response to reduced auxin concentrations in the main stem. As it is not required for bud activation, and indeed the timing of downregulation does not correlate robustly with the timing of initiation of rapid bud elongation, what, then, is the function of this reduced *BRC1* expression? If low stem auxin does indeed act in two distinct ways, promoting auxin transport canalization out of the bud, and downregulating *BRC1* expression, this creates what is essentially a coherent feedforward network motif. This type of regulatory circuit has been shown to increase robustness in switching mechanisms and to affect unidirectionally the timing of switching (Mangan and Alon, 2003; Shen-Orr et al., 2002). The effects of *brc1-2 brc2-1* mutation on bud auxin response are very interesting in this context. In a wild-type background, which according to our model would have both arms of the feedforward system, increased stem auxin delays bud activation, which eventually proceeds rapidly in a strong switch-like manner. By contrast, when one of the arms of the feedforward loop is removed, as in the *brc1-2 brc2-1* mutant background, increased stem auxin only weakly delays initiation of growth, but the switch-like behaviour of wild-type buds is attenuated to give a more gradual response (Fig. 4).

This system differs in several important respects from the well-studied feedforward circuits, which are microbial transcriptional on-off switches (Mangan and Alon, 2003; Shen-Orr et al., 2002). In the case of bud activation, the canalization mechanism is itself driven by a positive-feedback loop that provides switch-like behaviour and hysteresis, making reversibility difficult. Furthermore, active buds export auxin into the stem (Balla et al., 2011; Li and Bangerth, 1999; Morris, 1977; Thimann and Skoog, 1933), thereby closing a negative-feedback loop by re-establishing high stem auxin. Nonetheless, a dual acting system, such as the one we propose here, is likely to confer properties to the bud activation switch that are not possible with a unitary pathway, and this will be important to explore further.

### *BRC1* and strigolactone signalling

Integration of *BRC1-*mediated and auxin transport canalization-mediated bud regulation also allows integration of two current models for the mechanism of action of SL in bud control. There is strong evidence that SL regulates shoot branching by triggering depletion of PIN1 auxin exporters from the plasma membrane of cells in the shoot auxin transport network (Bennett et al., 2006; Crawford et al., 2010; Prusinkiewicz et al., 2009; Shinohara et al., 2013). This reduces the positive feedback in auxin transport canalization, making it more difficult for buds to activate.

In several dicot species, SL also upregulates the transcription of *BRC1*, providing another mechanism by which SL regulates bud activity (Braun et al., 2012; Dun et al., 2012, 2013; Mashiguchi et al., 2009). However, it is clear that this mechanism is not sufficient to explain SL-mediated bud inhibition. Consistent with results in pea (Braun et al., 2012), our results show that SL signalling mutants have more branches than *brc1-2 brc2-1* mutants, especially in conditions that increase the number of vegetative nodes (Fig. 7). Furthermore, this more discriminatory environment provides convincing evidence that triple mutants lacking *BRC1*, *BRC2* and SL signalling have a strongly additive phenotype (Fig. 7), resolving previously reported conflicting results in *Arabidopsis* (Aguilar-Martínez et al., 2007; Chevalier et al., 2014), and extending a similar result in pea that was considered to be surprising (Braun et al., 2012). In contrast to previous reports (Brewer et al., 2009; Braun et al., 2012), using more sensitive assays we demonstrate that *brc1-2 brc2-1* mutant buds are SL responsive in both whole plants and two-node bud-bud competition assays (Fig. 8). Interestingly, mutation in *brc1* is epistatic to *smxl678* mutation. Although this could be interpreted as demonstrating a requirement for *BRC1* in SL-mediated bud inhibition, this is inconsistent with our other results. Rather, this epistasis is easily explained by the bud activation threshold model. According to this model, *brc1* mutation increases branching in a wild-type background by a fixed amount by reducing the bud activation threshold. In the same way, *brc1* mutation in the *smxl678 max2* quadruple mutant background, which has wild-type levels of branching, increases branching by a fixed amount, producing branching levels equivalent to those in *brc1*.

Although SL can clearly act independently of *BRC1*, the constitutively low levels of *BRC1* expression in SL-deficient mutants, and the constitutively high levels in *smxl678*, suggest that *BRC1* is indeed a transcriptional target of SL signalling. This suggests that SL affects both arms of the proposed feed-forward loop in parallel, through effects on both PIN protein trafficking and *BRC1* transcription. Consistent with parallel action, *brc1* mutants have wild-type PIN protein accumulation and auxin transport mutants have wild-type *BRC1* expression (Bennett et al., 2016b). Thus, it seems likely that, like low auxin in the main stem, SL regulates bud activity via a coherent feedforward circuit involving effects on both *BRC1* transcription and auxin transport canalization.

Interestingly, in monocots, there is very little evidence that SLs act via transcriptional regulation of *BRC1* family members. For example, in rice, *FC1* does not appear to be transcriptionally regulated by SL, and SL mutants are highly branched despite wild-type levels of *FC1* expression (Arite et al., 2007; Minakuchi et al., 2010). Similarly, SL-deficient maize mutants have a highly branched phenotype, despite constitutively high *TB1* expression (Guan et al., 2012). If this does represent a consistent difference between monocots and dicots, it will be interesting to assess what the functional significance and evolutionary origin of the difference might be.

## MATERIALS AND METHODS

### Plant material and growth conditions

*Arabidopsis* Col-0 wild-type plants were used throughout. The following lines used have been previously described: *brc1-2*, *brc1-2 brc2-1* (Aguilar-Martínez et al., 2007); *lexa::BRC1* (González-Grandío et al., 2013); *d14-1* (Waters et al., 2012); *max2-1* (Stirnberg et al., 2002); *max4-5* (Bennett et al., 2006); *smxl6-4 smxl7-3 smxl8-1, smxl6-4 smxl7-3 smxl8-1 max2-1* (Soundappan et al., 2015); and *ft-10* (Yoo et al., 2005). Higher order mutant combinations were generated by crossing, using visible and PCR-based markers for genotyping.

Plants were grown on Levington's F2 compost in glasshouses with a temperature range of 15-24°C. Daylight was supplemented when necessary to ∼150 µmol photons m^−2^ s^−1^ to 16 h/8 h light/dark. For short-day conditions, plants were grown in Conviron growth chambers with white fluorescent tube lighting at ∼150 µmol photons m^−2^ s^−1^, 8 h/16 h light/dark, 22/18°C.

Assessment of branching responses to nitrate availability was as described by de Jong et al. (2014). Plants were grown in the glasshouse on a sand/Terra-Green mixture supplemented by *Arabidopsis thaliana* salts (ATS) (Wilson et al., 1990) containing either 9 mM or 1.8 mM nitrate.

Excised shoot explants were grown in Eppendorf tubes containing 1.95 ml ATS. The explants were transferred to Conviron growth chambers with white fluorescent tube lighting at ∼150 µmol photons m^−2^ s^−1^, 16 h/8 h light/dark, 22/18°C. The tubes were kept in trays containing wet filter paper and covered with propagator lids to reduce evaporation. For experiments with time-lapse photography, Conviron growth chambers with 24 h light were used.

Plants were grown axenically as described by de Jong et al. (2014). Seven seeds were sown per glass jar containing agar-solidified ATS medium supplemented with *rac*-GR24 dissolved in acetone or with solvent control.

### Bud growth assays

Auxin dose-response assays were carried out as described by Chatfield et al. (2000). Buds were held between two agar slabs in a Petri dish, allowing apical auxin treatments, with bud growth followed over time.

Non-axenic bud growth and gene expression assays were carried essentially as described by Ongaro et al. (2008). Shoot apices of glasshouse-grown plants bearing two or three leaves were excised 2-3 days after bolting and placed in Eppendorf tubes as described above. Explants were allowed to acclimatise for 3 days, which also allowed elongation of the main stem, enabling isolation of a single node by removing more basal nodes. For bud-bud competition assays, two buds were left on the explants. Explants were decapitated and/or were transferred to fresh tubes with appropriate supplements. Bud samples were collected for gene expression analysis, or bud was length measured as appropriate.

Basal hormone or gene induction treatments were carried out by supplementing the ATS solution with the appropriate hormone or inducer. Apical hormone treatments were carried out by applying lanolin paste to the decapitation site. 6-benzylaminopurine (BAP) was dissolved in DMSO and GR24 was dissolved in acetone. 1-Naphthalene acetic acid (NAA) was dissolved in ethanol for application in agar and in DMSO for application in lanolin. Where NAA was mixed with lanolin, 5 µl 6× Bromophenol Blue-based gel loading dye (NEB) was also added to assess mixing.

### Time-lapse photography

Silica microsphere beads (50 µm, Corpuscular) were stained with Bromophenol Blue and stuck using lanolin at the junction between the main stem and the bud stem, and where the bud stem meets the oldest bud leaf. Digital cameras (Nikon) with macro lenses and two extender focus tubes (Meike) were used to take images every hour for 24 h. The length of the bud stem (between the two beads) was measured using the manual tracking plug-in on Fiji.

### qRT-PCR

Pools of 15-20 buds were harvested and snap frozen in liquid nitrogen. Total RNA was isolated using the RNeasy Plant Mini Kit (Qiagen) and DNase treated with Turbo DNA-free Kit (Ambion) according to the manufacturers' instructions. RNA was quantified with a NanoDrop 1000 and 1 µg RNA was used for cDNA synthesis using Superscript II (Invitrogen) primed with OligodT according to manufacturer's instructions. Transcript levels were quantified relative to *UBQ10* (*UBIQUITIN 10*; AT4G05320) using SYBR Green (Bioline) with 10 ng cDNA in a 10 µl reaction volume on a Light Cycler 480 II (Roche). Expression levels were calculated using the ΔΔC_t_ method using C_t_ values calculated by the 2nd derivative maximum function of the Light Cycler 480 II software.

### Statistics

For parametric testing, Student's *t*-test was carried out after log transformation of the data where necessary. For non-parametric tests, the Wilcoxon pairwise rank-sum test was used. For censored data (i.e. where data collection ceased before all individuals had exhibited the phenomenon being measured), Kaplan–Meier survival analysis was used with log-rank tests. Holm-Bonferroni adjustments were made for multiple comparisons.
